# Evaluation and Optimization of Methods for Generating High-Resolution Retinotopic Maps Using Visual Cortex Voltage-Sensitive Dye Imaging

**DOI:** 10.3389/fncel.2021.713538

**Published:** 2021-09-21

**Authors:** Ori Carmi, Adi Gross, Nadav Ivzan, Lamberto La Franca, Nairouz Farah, Zeev Zalevsky, Yossi Mandel

**Affiliations:** ^1^Faculty of Life Sciences, School of Optometry and Vision Science, Bar-Ilan University, Ramat Gan, Israel; ^2^Faculty of Engineering, Bar-Ilan University, Ramat Gan, Israel; ^3^Department of Ophthalmology Vita-Salute San Raffaele University, Milan, Italy; ^4^Bar Ilan’s Institute for Nanotechnology and Advanced Materials (BINA), Bar-Ilan University, Ramat Gan, Israel

**Keywords:** retinotopic mapping, signal processing, cluster evaluation, visual cortex (V1), voltage sensitive dye imaging

## Abstract

The localization and measurement of neuronal activity magnitude at high spatial and temporal resolution are essential for mapping and better understanding neuronal systems and mechanisms. One such example is the generation of retinotopic maps, which correlates localized retinal stimulation with the corresponding specific visual cortex responses. Here we evaluated and compared seven different methods for extracting and localizing cortical responses from voltage-sensitive dye imaging recordings, elicited by visual stimuli projected directly on the rat retina by a customized projection system. The performance of these methods was evaluated both qualitatively and quantitatively by means of two cluster separation metrics, namely, the (adjusted) Silhouette Index (SI) and the (adjusted) Davies-Bouldin Index (DBI). These metrics were validated using simulated data, which showed that Temporally Structured Component Analysis (TSCA) outperformed all other analysis methods for localizing cortical responses and generating high-resolution retinotopic maps. The analysis methods, as well as the use of cluster separation metrics proposed here, can facilitate future research aiming to localize specific activity at high resolution in the visual cortex or other brain areas.

## Introduction

Recording activity from a large population of neurons serves as an important tool for studying numerous neural systems in general, and for exploring the basic mechanisms underlying the visual process in particular. Various techniques exist that attempt to estimate and classify the cortical responses from a noisy signal (Blumenfeld, 2010; Chemla and Chavane, 2010; Reynaud et al., 2011), each method has its own advantages and limitations. Electrophysiological studies have revealed that in mammals the localized stimulation of a specific retinal region (corresponding to specific locations in the field of view) induces a localized response in V1 and that there is an orderly representation of the visual field of view in the V1, the so-called retinotopic mapping (Hubel and Wiesel, 1959). Generation of retinotopic maps is of great interest for studying retinal-visual cortex processing, plasticity, circuitry, and for better understanding various pathologies (Hubel and Wiesel, 1959; Gross et al., 2019). The typical response in the visual cortex to localized retinal stimuli is activity that starts spatially at the retinotopic site of the visual stimulus (Bringuier et al., 1999; Roland et al., 2006; Gao et al., 2012) and then spreads over several square millimeters (Hubel and Wiesel, 1959; Grinvald et al., 1994; Michel et al., 2018). Retinotopic maps, depicting the cortical area corresponding to specific retinal (or visual field) locations, are usually generated by a variety of signal processing methods, all of which use prior temporal information about the response, noise, and the stimulus design (Blumenfeld, 2010; Reynaud et al., 2011; Omer et al., 2013). Many algorithms and methods for denoising and extracting the cortical responses exist in the literature, ranging from blank-subtraction and averaging (Chakraborty et al., 2007; Bollimunta et al., 2008), PCA/ICA (Cannestra et al., 1996; Maeda et al., 2001; Sornborger et al., 2003; Reidl et al., 2007; Omer et al., 2013) to linear models (Reynaud et al., 2011; Chemla et al., 2017).

Here, we evaluate and compare (both qualitatively and quantitatively) various analysis methods for extracting cortical responses, specifically aiming to localize the elicited cortical responses in order to generate the corresponding retinotopic maps. As a first step, we used simulated artificial data to initially evaluate various analysis methods. Then, we applied the same methods on extensive experimental data from voltage-sensitive dye imaging (VSDI) recordings of the rat’s visual cortex. Following the generation of the retinotopic maps, we used cluster separation metrics to quantify the resolution and accuracy of the retinotopic maps generated by the analysis methods.

## Materials and Methods

### General Approach

We implemented and compared seven different methods for analyzing the cortical responses elicited by visual retinal stimulation: (1) Average of Frames (AOF), (2) Multi-Parametric Thresholding (MPT), (3) Maximal Cross-Correlation Delay (T_max_), (4) Correlation to delayed responses (Corr), (5) Temporally-Structured-Component-Analysis (TSCA), (6) the Generalized Linear Model (GLM), and (7) a combination of GLM and TSCA. A detailed description of these methods is given below under “Seven methods for estimating individual cortical responses.” As a first step in implementing the different data analysis methods, we used simulated data for which the signal and noise (and the SNR) are known, enabling us to accurately quantify the ability of each method to extract cortical responses and generate retinotopic maps (see retinotopic map generation below). Then, we repeated these steps for experimental VSDI data recorded from the rat visual cortex. To estimate the cortical responses, the methods rely on a temporal prior information of the signal. The theoretical response curve, which is used by the TSCA, Tmax, Correlation, and GLM analysis methods (see below) as the temporal prior of the response (i.e., signal), was constructed as described in Supplementary Material the theoretical response curve section. The retinotopic maps generated by the different analysis methods were evaluated using statistical and cluster analysis methods.

### Simulated Data

**FIGURE 1:**
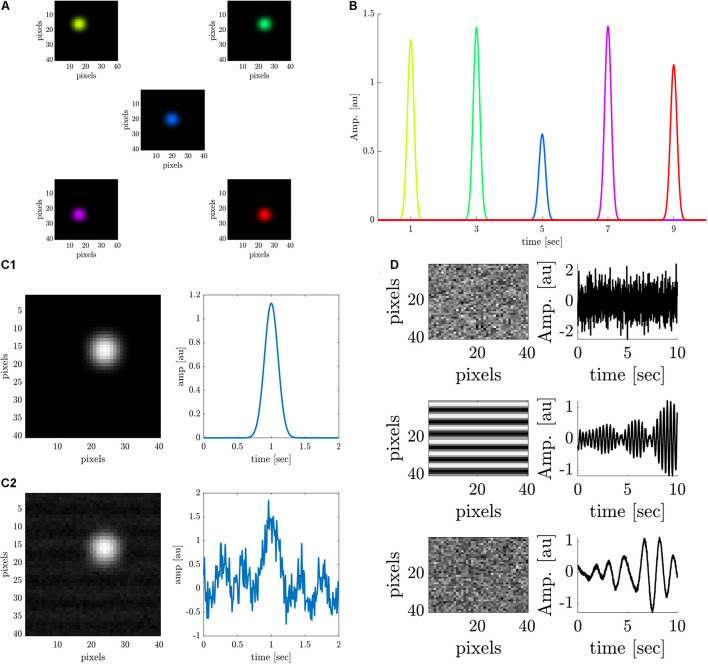
Data analysis on stimulated cortical data. **(A)** Simulated spatial cortical responses at 5 different locations generated at a rate of 0.5 Hz. **(B)** The temporal dynamics of the 5 simulated responses elicited at a rate of 0.5 Hz (corresponding to colors in **A**). **(C1,C2)** Upper row: A simulated cortical response without additive noise. Lower row: A simulated cortical response with additive noise (SNR = 1.5 dB). **(D)** Spatial and temporal components of the additive noise; upper row: Additive white noise; middle row: Additive periodic noise of 3 Hz; lower row: Additive periodic noise of 0.67 Hz.

We generated the simulated data of an ideal retinotopic mapping experiment, where visual stimuli are presented at different times and at different locations of the retina while the cortical responses are recorded. Accordingly, simulated cortical responses similar to experimental VSDI cortical responses (Gross et al., 2019) were generated at different cortical locations (Figure 1A). The simulated responses were generated at a rate of 0.5 Hz for a 10-s “experiment”, to represent the expected responses in our experimental setting, peaking every 2 s (at 1, 3, 5, 7, and 9 s) (Figure 1B), corresponding to the stimulus that elicited them. Each color represents the location of the response to a different retinal stimulus. Each simulated cortical response was a collective of pixels comprising a disc with a radius of 4 pixels, gaussian-filtered with σ = 2*pixels*. The temporal evolution of the signal was a gaussian curve with a random amplitude drawn from a uniform distribution between [0.5, 1.5], and σ = 1. Figure 1C1 shows a close-up of the spatial and temporal cortical response, before noise was added, which is detailed next.

Noise was added to the data to simulate white noise (random cortical activity) and periodic noise arising from heartbeat (3 Hz or breathing 0.67 Hz) (Lippert et al., 2007; Chemla et al., 2017). First, a constant value (DC) was added to the entire image, to mimic a constant noise level. Then, three noise sources were linearly added to the signals (Figure 1D), with specific spatial components: a random white noise (upper trace), a 2D cosine function along the y-axis (median trace), and additional random white noise (lower trace). The three corresponding temporal components of the noise are as follows:ϵ(*t*) gaussian white noise process N (0,σ), an almost periodic signal at 3Hz, and an almost periodic signal at 0.67 Hz. The almost periodic signals were created by adding sinusoidal signals in a 0.4 Hz bandwidth around the central frequency, with random amplitude and phase [both drawn from ϵ(*t*)]. Figure 1C2 shows a close-up of the spatial and temporal response polluted with additive noise (SNR = 1.5 dB).

Different theoretical signal-to-noise ratios (SNRs) were simulated, ranging from zero noise (infinite SNR) to an SNR of −30 dB. We repeated the simulation 100 times for each SNR to evaluate the performance of the six different analysis methods. In each repeated simulation, the signals and noise were randomly drawn from their corresponding distributions. Individual cortical responses were extracted by each of the different methods (see the detailed description below) and then were rescaled to Blumenfeld (2010). Finally, retinotopic maps were generated from the individual cortical responses (see *“Retinotopic Maps Generation”* below).

### VSDI Cortical Recordings

#### Animal Preparation

All experimental and surgical procedures were approved by the Bar-Ilan University Ethics Committee for animal research and were carried out in accordance with the ARVO guidelines for the Use of Animals in Ophthalmic and Vision Research. We used male and female 8–12-week-old (200–250 g) wild-type Long Evans rats. Animals were initially subcutaneously injected with Domitor (Medetomidine hydrochloride 1 mg/mL; 0.3 mg/100 g body weight, Orion Pharma, Finland) and put on isoflurane inhalation (1.2 mL/h), with a periodic addition of half the initial Domitor dose every 2 h. The animals’ temperature was maintained throughout the experiment (36.5–37.5°C) with a homeothermic blanket, controlled through feedback from a rectal probe.

All surgical procedures and dye staining were performed as described previously (Gross et al., 2019). Briefly, following anesthesia, the head was fixed using a custom-made steel chamber, attached to the skull above the right primary visual cortex (V1). A craniotomy (6 mm) was performed; then the dura was removed to expose V1. The exposed V1 was stained using a mixture of Artificial Cerebro-Spinal Fluid (aCSF) and a voltage-sensitive dye (RH-2080 1 mg/mL, a new modified version of the RH-1691 (Shoham et al., 1999) with only different substituents on the two-linked chromophores (Deneux and Grinvald, 2017); Optical Imaging, Rehovot, Israel) for 2 h. The brain tissue was kept hydrated throughout the entire procedure by replenishing the chamber with aCSF. Following the staining procedure, lukewarm agar was poured into the chamber and then sealed with a microscope cover glass for optimal optical transmission.

#### Visual Stimulation

**FIGURE 2:**
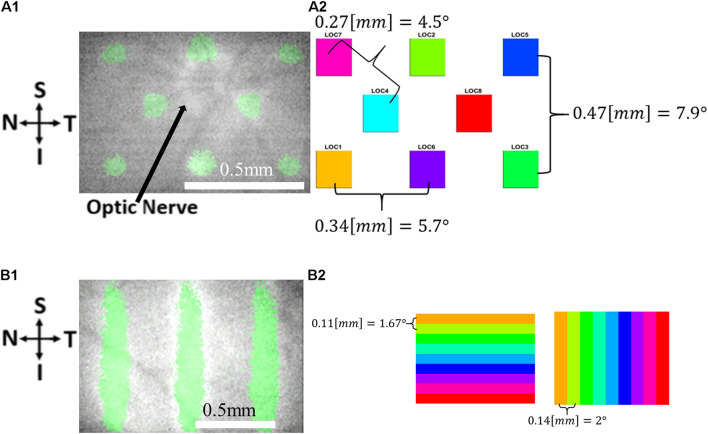
The visual stimuli used in the VSDI experiments. **(A)** Retinal imaging **(A1)** and a schematic drawing **(A2)** showing the 8 location stimuli. **(B)** Retinal imaging **(B1)** and a schematic drawing **(B2)** showing the 9 bar stimuli: Horizontal (left) and vertical (right) 9 bar stimuli.

Visual stimuli were projected using a costume-built projection system as previously described (Gross et al., 2019). The system consists of a Digital Light Processor (DLP) Digital Micrometer Device (DMD) projector (DLP LightCrafter 4500, Texas Instruments, Inc., Dallas, TX, United States) controlled by software and passive optical elements that project an image onto the retina of the rat. The projector light source is a green LED (520 nm). Five different visual stimuli were used for eliciting the cortical responses, as follows: three 100 μm (∼1.5°) micrometer width vertical bars with a horizontal spacing of 300 μm (comprising ∼4.5° field of view in the rat eye), a grid of 4 rectangles with a diagonal distance of 270 μm (∼4° field of view in the rat eye), a grid of 8 rectangles (Figure 2A1) at a diagonal distance of 270 μm (comprising ∼4° field of view in the rat eye), nine moving vertical bars (Figure 2B1) (horizontal distance ∼2°, velocity of 4°/s, a total retinal distance of 1.3 mm (∼18°)); and horizontal bars (vertical distance ∼1.67°, velocity 3.3°/s, a total retinal distance of 1 mm (∼15°).

To project the stimulus on the retina, the pupil was dilated through topical application of Mydramide (Tropicamide 0.5%, Fisher Pharmaceutical, Ltd.) and Efrin-10 drops (Phenylephrine HCL 10%, Fisher Pharmaceutical, Ltd.), after which a microscope cover glass (*d* = 13 mm) was mounted on the cornea, coupled by an ophthalmic gel, similar to previous publications (Mandel et al., 2013; Gross et al., 2019). Real-time imaging of the retinal stimulus was performed by a camera (DMK 33GP1300, The Imaging Source, Bremen, Germany), enabling the localization and focusing of the stimulus on the retina at a desired location with high precision throughout the experiment.

#### Image Acquisition

To record the fluorescence changes in the visual cortex, we used the setup previously established by our group (Gross et al., 2019). The cortex was excited with 630 nm light and the emitted fluorescence was collected at 665 nm, captured by a CMOS camera (Photonfocus, Switzerland, 12bit), at a rate of 100 Hz and resolution of 1,080 by 1,308 pixels (∼200 pixels/mm, thus capturing 5.39 mm by 6.53 mm of the brain). To reduce size on disk and render data analysis more computationally efficient frames were down-sampled by a factor of 4, resulting in ∼50 pixels/mm.

### Data Processing and Analysis

Following acquisition, VSDI data from 22 sets of VSD recordings from 7 different animals were pre-processed (see Supplementary Material *Data Pre-Processing*). Then, six different methods were implemented to localize the cortical responses to each retinal stimulus. A detailed description of each method is presented next. For all investigated methods and to ensure the validity of the comparison, we used constant parameters across the entire simulated data and the experimental data (see Supplementary Tables 1, 2). Additionally, as a seventh analysis method, we combined the TSCA and GLM methods. Data were then post-processed for denoising and scaling (described in Supplementary Material under *Data Post-Processing*) and retinotopic maps were generated from the responses of individual retinal stimuli (see the *“Retinotopic Maps Generation”* section below).

A qualitative and quantitative evaluation of the generated retinotopic maps was then performed in order to compare the seven different methods. To this end, we used statistical evaluation metrics and clustering separation metrics (the Davies-Bouldin and Silhouette index) to evaluate the ability of each method to extract cortical responses and generate a precise retinotopic map (see detailed descriptions in the section *Retinotopic Maps Cluster Evaluation*).

#### Six Methods of Localizing Cortical Responses

(1) Average of Frames (AOF)
  Through this technique, the cortical response is obtained by averaging the N acquired frames at the timing window where the response is expected. Given an expected response onset at *t*_*res*_(for example, 150 ms post-stimulus onset), the cortical response of this stimulus can be obtained by:
  (1) $$\;map=\;\frac{\sum_{i=0}^{N-1}Z(t_{res+i})}{N}$$
  where *Z*(*t*) is the frame at time *t* and *N* is the number of frames to average. Given *n* projected stimuli, this procedure can be repeated *n* times for each *t*_*res*,*k*_, *k* ∈ [1, *n*] value. Hence, one free parameter is chosen manually: (1) the number of frames to average from *t*_*res*_.
(2) The Multi-Parametric Thresholding System (MPT)
  The multi-parametric thresholding is based on a method recently reported by our group (Gross et al., 2019). Briefly, the cortical response is extracted by a multi-thresholding process and several criteria for each pixel are applied: First, pixels responding above a certain threshold value are selected. Next, the xth% of the pixels with the highest peak amplitude and for which the peak amplitude (the latency of the response) and return to baseline in limited predetermined time windows, are selected. As such, this technique has several free parameters that can be chosen: the absolute threshold value, the percentage of the highest peak amplitude to be isolated, the time window for which the peak amplitude must be in, and the baseline value and the time to return to it. These parameters are chosen by trial and error, starting at thresholds that are expected theoretically, and are adjusted until the optimal map is obtained.
(3) Maximal Cross-Correlation Delay (T_*max*_)
  This method is based on determining the stimulus that elicited the maximal response using cross correlograms and has been previously used to generate retinotopic maps to moving bar stimuli in the mouse visual cortex (Polack and Contreras, 2012). Briefly, a theoretical temporal reference curve, *g*(*t*), is generated with the shape of the expected cortical response (usually an alpha function is chosen, of the form *g*(*t*) = *ate*^−*bt*^*c*). Then, each pixel’s temporal response, *z*_*i*_(*t*), is cross correlated with *g*(*t*), to produce a cross correlogram delay function, defined as:
  (2) $$r\left(\tau \right)∼\int z_{i}\left(t\right)g\left(t-\tau \right)dt$$
  Each pixel’s cross correlation function peaks at a different delay τ_*max*_, i.e., ∀τ,*r*(τ_*max*_) > *r*(τ), corresponding to an activation elicited by a stimulus at a specific time (τ_*max*_ − Δ*t*, Δ*t* is the response latency + rise time). To avoid representing cortical regions (pixels) that did not produce a response, i.e., having a low correlation with *g*(*t*), and to obtain a better visualization, only pixels with a correlation peak above a certain threshold were considered as responsive and are selected to be represented on a map. Thus, this technique has two free parameters that can be selected: the theoretical response shape and the (response) threshold for maximal correlation.
(4) Correlation to Delayed Theoretical Responses (Corr)
  This technique is inspired by the above-described T_*max*_ method; however, instead of cross correlating each pixel’s temporal response, *z*_*i*_(*t*), to the theoretical reference curve *g*(*t*), each *z*_*i*_(*t*) value is correlated to a delayed version of *g*(*t*), *g*_*k*_(*t*) = *g*(*t* − *t*_*k*_). Assuming *n* different stimuli given at times *t*_*k*_, *k* ∈ [1, *n*], an *n* × 1 vector of correlations, *v*_*i*_(*k*), is calculated for each pixel *i* by:
  (3) $$v\left(k\right)=\frac{1}{T}z_{i}\left(t\right)g\left(t-t_{k}\right)$$
  where *T* is the total recording time. The advantage of this method over T_*max*_ is that whereas T_*max*_ sets a response threshold using the maximum value of *r*(τ), it does not consider the delay of the maximum correlation, which might not be consistent with the expected response time. The disadvantage of this method is that small changes in the latency of the response can greatly affect the values of *v*(*k*). The 2 free parameters that can be selected using this technique are the theoretical response, *g*(*t*), the shape, and latency.
(5) Temporally Structured Component Analysis (TSCA)
  This algorithm was introduced in Blumenfeld (2010); it is also used (Omer et al., 2013) for generating orientation maps in a behaving monkey. Generally, a raw optical signal reflects both the desired neuronal activity (signal) and the noise originating from neuronal sources (spontaneous activity) and non-neuronal physiological sources (e.g., heartbeat and breathing, environmental noises, e.g., electrical pollution, and noises common to the imaging signal). The main idea behind the TSCA algorithm is to find components that maximize the power originating from the signal while minimizing power arising from the noise by exploiting *a priori* information about the temporal characteristics of the signal and noise. The advantage of TSCA over principle component analysis (PCA) is that PCA performs poorly in terms of separating the signal from the noise (Blumenfeld, 2010). In this case, the principal components are “non-interpretable” and reflect non-sensical mixtures of signal and noise.
  The objective function to be maximized in the TSCA method is defined as a weighted sum of the expected power of the signal and noise: $m\left(\psi \right)=\gamma_{x}s_{x}^{2}\left(\psi \right)+\gamma_{y}s_{y}^{2}\left(\psi \right),$ where $s_{x}^{2}\left(\psi \right)\&s_{y}^{2}\left(\psi \right)$ are the expected signal power and the noise projected on the component, respectively, and γ_*x*_ and γ_*y*_ are the weights assigned for the signal and noise powers, respectively. The first assumption of the TSCA algorithm is clear from this objective function: the additivity of the signal and noise. A quadratic estimator of the form ${\hat{m}}_{Q}\left(\psi \right)=\psi^{t}ZQZ^{t}\psi^{t}$ is proposed, where *Z* is the multidimensional signal and *Q* is an arbitrary symmetric *T* × *T* matrix. The solution to this maximization problem lies in the eigen-decomposition of the matrix *M* = *ZQZ^t^*, where *Q* is selected as the minimal (Frobenius) norm solution to the linear system of equations:
  (4) $$<Q,C_{x}^{i}\geq \gamma_{x}tr\left(C_{x}^{i}\right),<Q,C_{y}^{i}\geq \gamma_{y}tr\left(C_{y}^{i}\right)$$
  where *Q*, which minimizes the Frobenius norm, is a linear combination of *C*_*x*_ *and* *C*_*y*_, the (known) correlation matrices of the signal and the noise, respectively (see Blumenfeld, 2010) for a comprehensive derivation of *Q*). The eigenvalues of the eigenvectors ψ_*i*_ equal the estimate of *m*(ψ); hence, ψ_*i*_, with the highest eigenvalues, maximizes the objective function. Two more assumptions arise from this: (1) linear independency exists between the sets of correlation matrices and (2) the availability of enough data such that the estimator is close enough to its asymptotic value. Given that the stimulation timings and noise artefacts are known, one can obtain prior temporal knowledge about the signal and noise and construct *C*_*x*_ & *C*_*y*_. The response shape (for a stimulus given at *t=0*) with random amplitude *r* is defined by a fixed function *rg*(*t*); usually an alpha function, *g*(*t*) = *ae*^−*bt*^ + *c*, is chosen. We carried out TSCA for each response at times *t_k_*, i.e., *g*_*k*_(*t*) = *g*(*t* − *t*_*k*_). The autocorrelation function *C_x_* is given by [*C*_*x*_]_*jk*_ = *g*_*k*_(*j*)*g*_*k*_(*k*). Regarding the noise autocorrelation matrices, *C_y_*, we used the identity matrix to account for the influence of white noise in the data, and we created almost periodic Toeplitz matrices to account for the stationary oscillatory noises (see Omer et al., 2013) for a full calculation of these matrices. Each use of TSCA reveals spatial components that maximize the objective function, with the signal defined as *g*_*k*_(*t*) for each iteration, i.e., the cortical response map corresponding to the stimuli given at time *t_k_*. The free parameters to be selected with this method are γ_*x*_ *and* γ_*y*_ and *C_x_* and *C_y_*.
(6) Generalized) Linear Model Decomposition (GLM)
  This technique uses a linear model (LM) that enables one to denoise and extract the relevant signals of neuronal activity dynamics (Reynaud et al., 2011). Each pixel is a time series with a length of *T*, *y*(*t*), *t* ∈ [0, *T*], which is decomposed into a finite weighted sum of regressors (or predictors) *x*_*k*_(*t*) with their respective weights β_*k*_, such that:
  (5) $$y\left(t\right)=\sum_{k}\beta_{k}x_{k}(t)r(t)$$
  where *r*(*t*) are the residuals, drawn from a (white) gaussian distribution with zero mean. Linear regression minimizes the squared error *J*(β) = ∑_*i*_(*y*_*i*_ − β^*t*^*X*_*i*_)^2^; hence, it is a convex optimization problem with a unique solution that lies at gradient zero, β = (*X^t^X*)^−1^*X^t^*. In order to efficiently use a linear model, the set of regressors *X* needs to accurately describe the observed time series and leave out only white residuals. This method first assumes the linear additivity of the signal and noise components. Cortical signals usually have non-neural periodic components, arising from breathing and heartbeat. These components have a fundamental frequency and can be expressed with a Fourier series. The Fourier coefficients can be estimated by using the complex exponentials as regressors and are then subtracted from the entire signal to denoise it from periodic noise. The LM decomposition can be used as a denoising step, and its superiority over blank subtraction has been previously demonstrated (Reynaud et al., 2011). If all “deterministic” components are regressed and subtracted from the recorded signal, only random white (gaussian) noise should remain. This noise can be attributed to spontaneous neural activity and other random non-physiological noises. In addition to denoising, one can also use the response curves at different times (corresponding to the expected response time after different stimuli) as regressors and use their corresponding coefficients to estimate individual cortical responses to every stimulus. The free parameters that can be chosen using this method are the regressors for the signal and noise components.

#### Combining Methods

We tested several combinations of the above-described methods to achieve better localization and increased SNR. The clearest retinotopic maps, both qualitatively and quantitatively, were obtained by combining the first denoising data with GLM and then applying TSCA analysis. This combined processing can be parameterized by setting *C_y_* (the noise covariance matrix of TSCA) to only be the identity matrix, $C_{y}\left(i,j\right)=\{\begin{array}{ll} 1, & i=j\\ 0, & otherwise \end{array}$, since after applying GLM, only white noise should remain in the signal. A more detailed explanation of how we attempted to combine the methods as well as an example of the combination of AOF and Corr with TSCA (separately) can be seen in Supplementary Material under *combining methods*.

#### Retinotopic Map Generation

Following the extraction and localization of the cortical response from each retinal stimulus by the seven above-mentioned analysis methods, the localized responses were post-processed (see Supplementary Material) and then combined to generate the retinotopic map in which each pixel was then associated with a specific retinal stimulus. Each pixel received a (likelihood) score for each specific stimulus and the stimulus with the highest score (i.e., the stimulus that elicited the maximum response in the pixel) determined the stimulus associated with the pixel for generating the retinotopic map. At the end of this analysis, each pixel is defined by two characteristics: the stimulus associated with it (represented by the hue) and the score (represented by the value). For better visuality, only pixels with maximum scores (values) above the 90th percentile of all the maximum scores were selected and displayed. Finally, the retinotopic maps were post-processed to remove (salt) noise and then scaled.

#### Retinotopic Mapping Evaluation Metrics

##### Statistical Evaluation Metrics of Retinotopic Maps (Simulation Only)

In a simulation study the correct or expected response is readily known; therefore, performance evaluation can be easily performed using several straightforward statistical methods. We used six different methods, namely, Mean Square Error (MSE), Peak-SNR (PSNR), the Contrast-to-Noise Ratio (CNR), the Mean Structure Similarity Index (MSSIM), the Correlation Coefficient (CC), and the Correlation Parameter (CP). These metrics were introduced in Wang et al. (2004) and Salinas and Fernández (2007), and are detailed in Supplementary Material.

##### Cluster Analysis of Retinotopic Maps (Simulation and Experimental Data)

As opposed to simulation studies, no *a priori* knowledge of what the “correct” retinotopic map is in experimental data is available. We therefore considered the cortical responses to various stimuli at each retinotopic map as clustered responses and evaluated the separation of the cortical responses using cluster analysis. First, we evaluated the retinotopic maps using two cluster analysis methods, namely, the Davies-Bouldin Index (DBI) (Davies and Bouldin, 1979) and the Silhouette Index (SI) (Rousseeuw, 1987). The DBI method uses the clusters’ scatter and (centroid) distances to evaluate the similarity. The SI calculates the similarity of each data point to its own cluster as opposed to all other clusters. The expected number of clusters and cluster sizes are known; thus, we propose an adjusted Silhouette and DBI; every cluster is penalized according to its size and every map is penalized according to the number of activity centers (clusters) extracted. Full details regarding the adjusted indices are described in Supplementary Material (Equations s10 and s11) under *Cluster Separation Metrics*.

## Results

### Simulation Study

**FIGURE 3:**
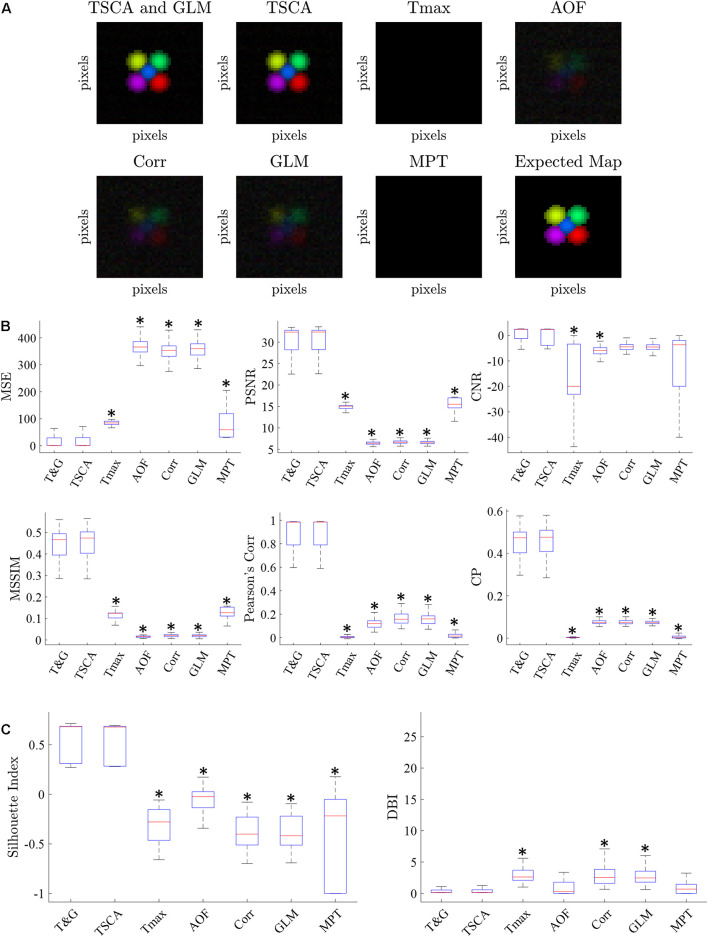
Simulated data retinotopic maps. **(A)** An average “retinotopic map” generated by each method, following 100 repetitions of simulated stimuli with an SNR = –10 dB. **(B)** The six statistical performance evaluation measures of the generated retinotopic maps presented in **(A)**. MSE, Mean Square Error; PSNR, Peak Signal-to-Noise Ratio; CNR, Contrast-to-Noise Ratio; MSSIM, Mean Structure Similarity Index; CC, Correlation Coefficient; CP, Correlation Parameter (CP). ^∗^ Significantly Different than the TSCA and TSCA & GLM methods (*p* < 0.05). **(C)** Cluster evaluation metrics of the generated retinotopic maps presented in **(A)**. The Silhouette index (left) and DBI (right). Significantly Different than the TSCA and TSCA and GLM methods (*p* < 0.05).

Figure 3 shows a characteristic cortical map generated by averaging 100 repetitions of simulated data with an SNR of −10 dB. The retinotopic maps generated by each of the seven analysis methods are compared to the expected map (lower right). Figure 3A shows representative average retinotopic maps generated by each of the above-described methods, and the combined TSCA&GLM (T&G). A qualitative evaluation shows that all methods except MPT and T_max_ could generate distinctive retinotopic maps from the noisy signal. However, TSCA and TSCA&GLM reproduced the maps with the highest SNR and contrast.

Figure 3B presents the results of the six statistical evaluation metrics used for a quantitative comparison of the maps generated by the seven methods for an SNR = −10 dB. TSCA and the combined (TSCA&GLM) methods significantly outperformed the other methods in extracting the response (a multiple comparison *t*-test with Tukey-HSD, *p* < 0.05) in all metrics except the CNR. The combined method did not significantly perform better than TSCA alone. The statistical measures for a range of SNR (5–10 dB) are detailed in Supplementary Figure 3. An additional comparison between the retinotopic maps generated by the seven methods was obtained using clustering separation metrics [the Silhouette Index (SI) and the David Bouldin Index (DBI)], as is shown in Figure 3C. In agreement with the statistical evaluation metrics, the TSCA and the combined TSCA&GLM methods achieved a significantly better clustering performance with significantly higher SI compared to all the other methods, and a significantly lower DBI compared with T_max_ GLM and Corr (a multiple comparison *t*-test with Tukey-HSD, *p* < 0.05). There was no statistically significant difference between TSCA and the combined TSCA&GLM. Both TSCA and the combined TSCA&GLM have a positive median silhouette index (0.67 and 0.69, respectively), indicating that most of the pixels were correctly mapped to their corresponding responses. Moreover, these values also approach the median SI of the simulated signals of 0.86 (Figure 1A). Both methods have the lowest median DBI (0.18 and 0.17, respectively), which is just above the ideal DBI of the simulated signals of 0.14 (Figure 1A), suggesting that in general, the clusters are well separated. In contrast, the T_max_ and MPT methods failed to extract the response from the noise and thus barely generated a clear retinotopic map. This failure is probably because these methods do not attempt to denoise the signal. Thus, for the MPT method with the low SNR data the peak amplitude deviates randomly from the expected response latency, therefore affecting the calculated score of every pixel assigned to a specific stimulus. Similarly, for the Tmax method, a noisy signal leads to erroneous cross-correlation values of many pixels, therefore leading to noisy, low scores, and poor retinotopic mapping. Interestingly, the Corr and AOF methods performed slightly better in analyzing noisy data compared with MPT and T_max_ because the theoretical response curve and timing of the response are known.

**FIGURE 4:**
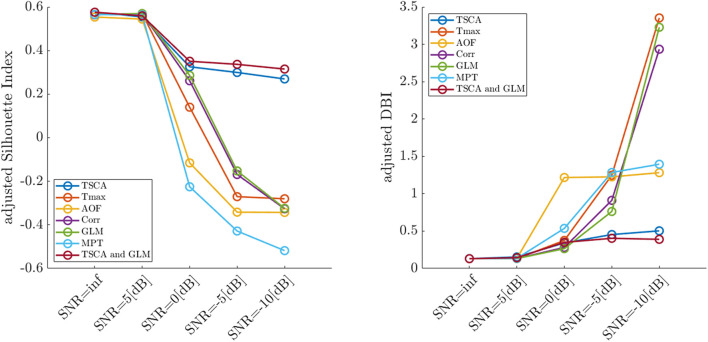
Average values of the adjusted Silhouette Index (left) and the adjusted DBI (right) of retinotopic maps generated by the seven analysis methods for simulated cortical responses with decreasing signal-to-noise ratios.

Figure 4 depicts the average adjusted SI (left) and the adjusted DBI (right) calculated for each SNR from 100 simulation trials. Reducing the signal-to-noise ratio was associated with decreased cluster separation performance (decreasing SI and increasing DIB), thus validating the use of these metrics for quantifying the retinotopic maps. The combined TSCA&GLM and the TSCA methods resulted in similarly high performance compared to other methods in terms of the cluster separation metrics, particularly under low SNR conditions.

To evaluate the effect of combining Corr or AOF with TSCA, we performed further simulation analysis, as described in the *Combining Methods* section in Supplementary Material. We found that the combination of either of these methods resulted in a degradation of both the statistical and the cluster analysis measures compared to the TSCA method alone (Supplementary Figure 4). Note that TSCA’s computation time (reduced time, eigen-decomposition of a subset of eigenvectors instead of *ZQZ^t^*) is at least two orders of magnitude longer than all other methods (Table 1). Thus, though TSCA (and TSCA and GLM) outperformed all other methods, it is not suitable for real-time applications.

**TABLE 1:** Computation times of the seven analysis methods for a single repetition of simulation.

	TSCA	T_*max*_	AOF	Corr	GLM	MPT
Computation time (ms)	16,582.2 ± 1.25	222.45 ± 43.2	1.28 ± 0.27	2.57 ± 0.64	84.46 ± 8.64	386.17 ± 37.89

### Experimental Data

**FIGURE 5:**
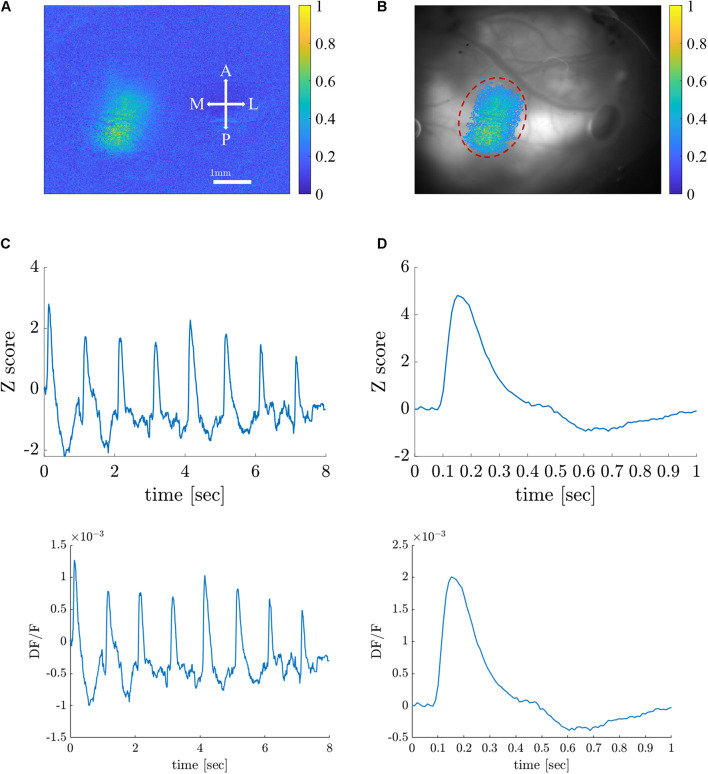
Visual cortex responses to short visual stimuli. **(A)** A color map representation of the cortical response amplitude (normalized Z-score) induced by visual stimuli. Scale bar and map orientations are overlaid. A, anterior; P, posterior; M, medial, and L, lateral. **(B)** Image of the brain overlaid with corresponding pixels in the visual cortex ROI (dashed red line). **(C)** A raw (z-scored) VSD signal in response to 8-grid stimuli given at a rate of 1 Hz in the ROI. **(D)** Characteristic average response to a single stimulus.

**FIGURE 6:**
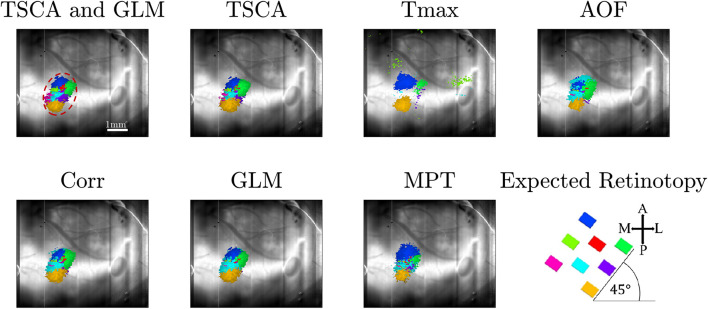
Retinotopic maps generated by the seven methods following a VSDI recording of cortical responses to 8-location grid retinal stimuli. Only pixels with score values above the 90th percentile of all the pixels’ maximum scores are presented. The scale bar shown in the top left panel is 1 mm. The cortical orientations are seen in the bottom right figure, along with the expected cortical response (A, anterior; P, posterior, and M, medial; L, lateral). Each color (hue) represents the cortical response location of the matched retinal stimuli.

**FIGURE 7:**
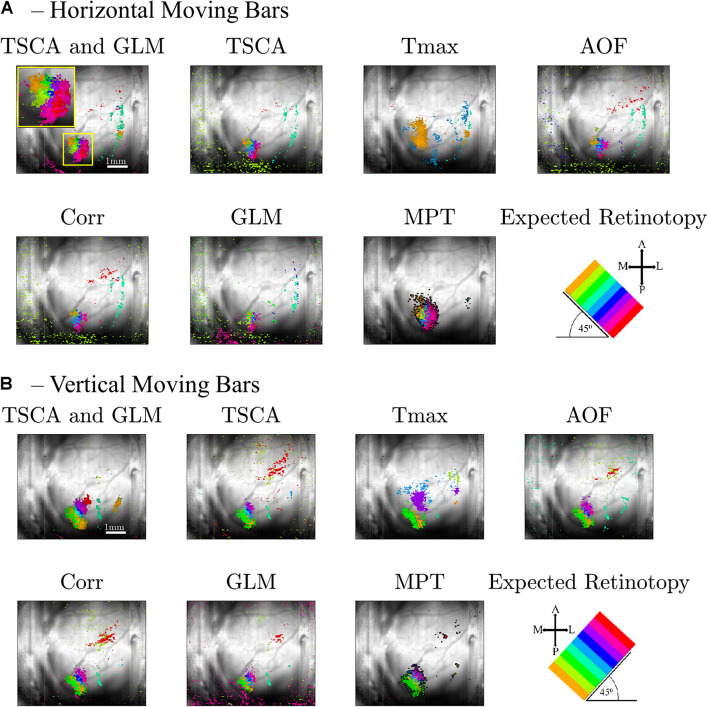
Retinotopic maps generated by the seven methods following a VSDI recording of cortical responses to high-resolution retinal stimuli of a 9-location moving (**A**, horizontal, **B**, vertical) bar. Only pixels with score values above the 90th percentile of all the pixels’ maximum scores are presented. The scale bar shown in the top left panel is 1 mm. The cortical orientations are seen in the bottom right figure, along with the expected cortical response (A, anterior; P, posterior; M, medial, and L, lateral). Each color (hue) represents the cortical response location of the matched retinal stimuli.

As a first step toward generating retinotopic maps, we evaluated the visual cortex response to 8-location grid short (20 ms) stimuli projected at a rate of 1 Hz. Figure 5A shows the maximum normalized Z-score (see Supplementary Material *Data Pre-Processing*) of each pixel throughout the entire experiment. Supplementary Figure 1 shows the dF/F signal. Only pixels with a maximum value above the 90th percentile of all values are selected and presented in the map. Figure 5B shows an image of the brain, overlaid with the selected pixels. The size of the region of interest (ROI) (denoted by a red dashed line) is 310×120μm. Figure 5C shows the average response of the ROI throughout the entire 8-s stimuli. Figure 5D depicts the average response of the ROI to a single stimulus duration of 1 s), which greatly resembles the theoretical characteristic response used in analyzing the experimental data (see Supplementary Figure 2). Next, we generated retinotopic maps for the 8-location grid retinal stimuli (see Figure 2A1) using the 7 analysis methods (described in section “Materials and Methods”). Figure 6 depicts a representative map where each color represents the location of the response to the matched retinal stimuli. As shown in this example of a low-noise experiment, all seven methods extracted the responses well enough to generate a suitable retinotopic map. All retinotopic maps exhibited the expected counter-clockwise rotation of the cortical respones, compared with the retinal stimulus locations (shown in the lower right panel), in agreement with previous retinotopic mapping experiments (Gias et al., 2005). Qualitative evaluation suggests that TSCA&GLM and TSCA produce retinotopic maps with the clearest distinction between cortical responses to different retinal location stimuli. Specifically, the responses in the center of the ROI (teal and red) are better represented in the combined TSCA&GLM map than with maps generated by other analysis methods. This is probably because both the TSCA and GLM methods are not biased toward stronger responses and thus can better localize the cortical response and consequently correctly associate the pixels with the corresponding retinal stimulus. Additionally, qualitative evaluation (Supplementary Figure 6) revealed that the combined method generated a stable and consistent retinotopic map following 4 repetitions of the same experimental sessions in the same animal. In contrast, other analysis methods showed a gradual deterioration in map quality, arising from both physiological and physical (e.g., dye bleaching) factors, causing a decreased signal-to-noise ratio as the experimental sessions progressed. Examples of retinopic maps generated in response to 9 (horizontally and vertically) bar stimuli are shown in Figure 7. Each color represents the location of the cortical response to the matched retinal stimuli in Figure 2B1. In this experiment, the retinal space between the retinal stimuli was significantly smaller than the 8-location stimuli (140 μm vs.270 μm), making the retinotopic mapping even more challenging. It should be mentioned that the resolution of this stimulation is still within the rat’s visual acuity of 1 CPD (Prusky et al., 2000; Lorach et al., 2015). Additionaly, this specific experiment contained significant surrounding noise, i.e., high energy noise outside the area of the responses (ROI). Similar to the results obtained for the 8-location stimulus paradigm, qualitative evaluation of the maps (Figures 7A,B) for this noisy signal revealed that GLM denoising combined with TSCA seems to outperform the other extraction methods, showing a map that corresponds relatively well to the expected response (the lower right Figure). All other analyses generated poor mapping of this high-resolution experiment.

**FIGURE 8:**
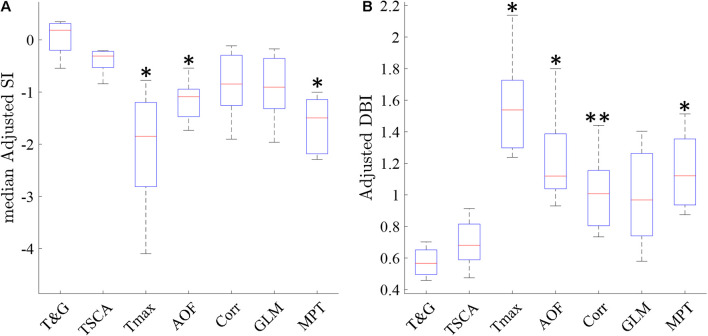
Cluster metrics evaluation of the analysis methods. **(A)** A Median Silhouette Index of the entire map. ^∗^ Significantly different compared with TSCA and TSCA & GLM (multiple comparison *t*-test with Tukey-HSD, *p* < 0.05). **(B)** A Davies-Bouldin Index of the entire map. ^∗^ Significantly Different than the TSCA and TSCA & GLM methods. ^∗∗^ Significantly different than the TSCA and GLM methods (multiple comparison *t*-test with Tukey-HSD, *p* < 0.05).

Following the qualitative analysis described above, we performed a quantitative analysis by examining the cluster separation ability of the seven methods using the SI and DB indices as described in section “Materials and Methods.” Figure 8 shows a box plot of the median adjusted SI and the adjusted DB indices for the maps generated by the seven methods in all 22 experiments. TSCA and TSCA&GLM showed the highest average adjusted SI (−0.43 ± 0.21 and 0.09 ± 0.27 for TSCA and TSCA&GLM, respectively) and the lowest adjusted DBI (0.7 ± 0.19 and 0.61 ± 0.12 for TSCA and TSCA&GLM, respectively), highlighting their superior performance in separating the response clusters, and thus enabling the generation of the most precise retinotopic maps. The cluster separation metrics of these two methods are better than all other methods; this difference is statistically significant compared with AOF, T_max_, and MPT (multiple comparison *t*-test with Tukey-HSD, *p* < 0.05).

**FIGURE 9:**
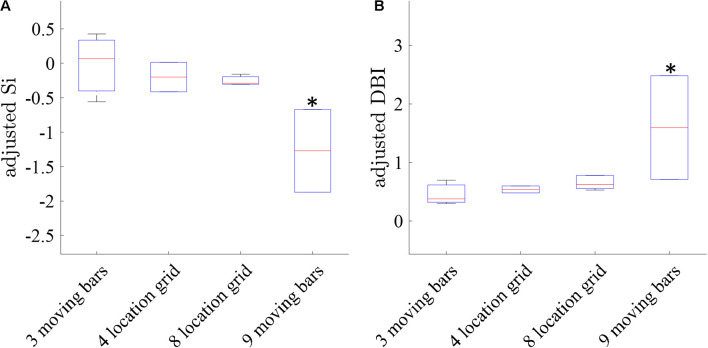
Comparison of the clustering separation metrics in the 3, 4 and 8-location grid and the more challenging 9-moving bars. Retinotopic maps in this comparison were performed by the combined GLM and TSCA analysis method. **(A)** The median Silhouette Index of the maps **(B)** The Davies-Bouldin Index of the maps. ^∗^ Significantly different than the 3 moving bars, 4 grid, and 8 grid stimulations (*p* < 0.05).

Finally, we compared the clustering separation metrics (SI and DBI) obtained for the maps induced by the four types of stimuli with increasing spatial resolution (and therefore more challenging to generate retinotopic maps) as follows: 3-bars, 4-grid, 8-grid, and 9-bars. Figure 9 shows that the clustering performance for the 3-bars, 4-grid stimuli, and 8-grid stimuli shows statistically significant (*t*-test *p* < 0.05) better cluster separation (average adjusted DBI = 0.46, 0.55, and 0.75, average adjusted SI = −0.09, −0.19, and −0.38 of 3 moving bars, 4-grid and 8-grid, respectively), compared with the more challenging 9-bars stimuli (average adjusted DBI = 1.59, average adjusted SI = −1.27), as expected. Similarly to the results obtained for the simulation data (Figure 4), these experimental results further support the validity of our method developed for the cluster analysis of retinotopic mapping.

Characteristic Retinotopic maps generated for three vertical bar stimulations and four location stimulations are shown in Supplementary Figure 6.

## Discussion

Functional mapping in the brain requires an accurate estimation of the cortical activity from a noisy biological signal. Similarly, the ability to accurately localize the discrete areas of activation in the visual cortex following visual stimulation at different locations is vital for successful high-resolution retinotopy. In this study, we present a thorough comparison of seven methods (six individual methods and one combined method) for extracting cortical responses from experimental VSDI recordings from rats’ visual cortex in response to various visual stimuli. As a preliminary step, we computer-simulated responses from the visual cortex. Following the extraction of the cortical responses, we generated retinotopic maps at high resolution by assigning each pixel to a specific visual stimulus. The retinotopic maps’ accuracy and resolution were then evaluated using six statistical metrics (for the simulation data) and two additional cluster separation metrics (for both the simulation and experimental data).

Our study clearly shows that TSCA and the combined TSCA&GLM analysis localized the cortical response areas most precisely, both in the simulation and experimental data. This was shown both qualitatively by examining the extracted retinotopic maps in the simulation (Figure 3A) and experimental data (Figures 6, 7), and quantitatively using statistical and cluster separation metrics (Figures 3B,C,4,8).

The superior performance of the TSCA analysis probably arises from two main properties of the algorithm: a good separation of the signal from the noise, as shown in Omer et al. (2013), and robustness to errors in the (assumed) temporal structure of the data and transformations to the data (e.g., adding DC) (Blumenfeld, 2010).

Linear Model Decomposition has been shown to be efficient in denoising images (Zheng et al., 2001) and VSDI signals (Chemla and Chavane, 2010; Reynaud et al., 2011; Chemla et al., 2017). In our study, GLM applied separately performed moderately in localizing the cortical response area, leading to the generation of lower resolution retinotopic maps. However, as a denoising step prior to performing TSCA (in the combined GLM&TSCA method), it slightly reduced the maps noise with some improvement (not statistically significant) in the cluster analysis measures. Quantitatively, both TSCA and the combined TSCA&GLM method showed statistically better cluster separation (Figure 8) than all other methods.

Indeed, because TSCA approach is less sensitive to changes in the characteristics of the physiological response and experimental noise, our results clearly show the superiority of this method over others across all experiments.

T_max_, which was previously reported for generating retinotopic maps (Polack and Contreras, 2012), seems to perform the worst of all the analysis methods used here, showing a bias toward strong responses and therefore failing to localize the weaker ones. Since T_max_ is based on cross correlating the VSDI signal with a theoretical response curve, in low SNR conditions, random delays may produce higher (random and incorrect) correlations. Hence, correlating each pixel with a pre-defined, delayed response curve better locates the responses (and at a lower computation time). Indeed, our evaluation showed that the Corr analysis method, which is proposed here as an alternative to T_max_, benefits from the temporal priors of the signal and therefore performs better than T_max_. However, since it does not attempt to denoise the signal simultaneously, its estimation of cortical activity is still not as accurate as the combined GLM and TSCA methods.

As is well known, averaging signals, both temporally and spatially, improves the SNR; similarly, averaging frames (AOF) around the peak of the cortical response is a standard method for enhancing images and improving SNR. However, it has a clear disadvantage; since the response propagates in the cortex as a point-spread function (Hubel and Wiesel, 1959; Grinvald et al., 1994), averaging more frames would hamper localizing the response and consequently, it would decrease the quality of the retinotopic maps.

Lastly, although MPT was reported by our group as a method for generating high-resolution retinotopy (Gross et al., 2019), it has the largest number of free parameters that can be chosen; therefore, it is very sensitive to changes in these parameters. MPT is mainly sensitive to changes in absolute parameters (i.e., ones that are not calculated from the signal) and to linear transformations to the data (e.g., a higher gain in the fluorescence caused by a strong stimulus) causing the generated retinotopic map to be less accurate. This can be clearly seen in Supplementary Figure 5. In addition, the need for tweaking its (many) parameters iteratively makes it difficult to correctly compare the results across different experiments and even in different trials.

The quality of the generated retinotopic maps was evaluated by the ability of each analysis method to create a localized area of cortical activity for each specific visual stimulus, i.e., that can be clearly distinguished from cortical areas activated by other stimuli. To this end, we used, for the first time, to the best of our knowledge, cluster separation metrics (DBI and SI) for evaluating the retinotopic maps. Both metrics showed a decline in performance when the signal-to-noise ratio is reduced in the simulated data (Figure 4). Furthermore, the performance analysis agreed with the quantitative evaluation and was further supported in experimental data where better performance was found for good retinotopy (the lower resolution retinal stimuli, Figure 2A2) compared with poor retinotopy (the 9-location moving bar with higher retinal stimulation resolution, Figure 2B2). The use of thresholding with these metrics can be employed to indicate whether a true separation between two areas of cortical activity exists in the visual cortex.

In a broader perspective, retinotopic mapping is just an example of cortical functional categorization task. Thus, the cluster analysis presented here can facilitate future studies seeking to evaluate the performance of categorizing brain areas or it can be used as a quantitative separation measure of functional brain areas other than V1.

Additionally, the analysis methods implemented and compared here have already been used in other imaging techniques (Reynaud et al., 2011) and for different analysis tasks (Polack and Contreras, 2012; Omer et al., 2013); therefore, they are suitable for extracting cortical responses or denoising signals for various techniques (such as fMRI, EEG, or MEG) and experimental paradigms. In the current study we suggest that the combined TSCA and GLM analysis can successfully denoise functional brain activity and can be used in the future for other functional recordings.

In conclusion, we extensively evaluated the performance of seven analysis methods for extracting cortical activity elicited by visual stimuli to generate retinotopic maps. Using cluster separation metrics, we found that TSCA and TSCA combined with GLM, exhibited the most superior performance in generating retinotopic maps from low signal-to-noise cortical activity obtained in response to high-resolution retinal stimulation. These combined methods can be further used to analyze many other cortical activities recorded in neuroscience research. Furthermore, the cluster separation analysis used for evaluating the map quality can be used in similar studies.

All the software code was written in MATLAB R2019b and is publicly available in “github”: https://github.com/oricarmi/VSDI_MATLAB_COMPARISON.git.

## Study Limitations

One potential limitation of the current research is the use of non-awake animals with variation in the anesthesia level greatly affecting brain activity. In addition, the generation of high resolution retinotopic maps can be limited by scatter arising from the retinal tissue.

## Data Availability Statement

The raw data supporting the conclusions of this article will be made available by the authors, without undue reservation.

## Ethics Statement

The animal study was reviewed and approved by Bar-Ilan University Ethics Committee for animal research.

## Author Contributions

OC and YM: conceptualization. OC, AG, NI, NF, and YM: methodology. AG, NI, OC, and LF: conducted experiments (data collection). OC and YM: writing—original draft. YM, NF, and ZZ: writing—review and editing. OC and NF: visualization. YM and ZZ: supervision and funding. All authors contributed to the article and approved the submitted version.

## Conflict of Interest

The authors declare that the research was conducted in the absence of any commercial or financial relationships that could be construed as a potential conflict of interest.

## Publisher’s Note

All claims expressed in this article are solely those of the authors and do not necessarily represent those of their affiliated organizations, or those of the publisher, the editors and the reviewers. Any product that may be evaluated in this article, or claim that may be made by its manufacturer, is not guaranteed or endorsed by the publisher.
