# Supplementary material for: Evaluation and Optimization of Methods for Generating High-Resolution Retinotopic Maps Using Visual Cortex Voltage-Sensitive Dye Imaging
Source: Front Cell Neurosci. 2021 Sep 21;15:713538. doi: 10.3389/fncel.2021.713538 (PMC8490879; doi:10.3389/fncel.2021.713538)
Supplement: Supplementary file 1 [file Data_Sheet_1.PDF]

Supplementary Material

Evaluation and Optimization of methods for generating high-resolution retinotopic maps using visual cortex voltage-sensitive dye imaging -

Ori Carmi[1,3], Adi Gross[1], Nadav Ivzan[1], Lamberto La Franca[1,4], Nairouz Farah[1], Zeev Zalevsky[3], and Yossi Mandel[1,2*]

[1] *Faculty of Life Sciences, School of Optometry and Vision Science, Bar-Ilan University, Ramat Gan 5290002, Israel*

[2] *Bar Ilan's Institute for Nanotechnology and Advanced Materials (BINA), Bar-Ilan University, Ramat Gan 5290002, Israel*

[3] *Faculty of Engineering, Bar-Ilan University, Ramat Gan 5290002, Israel*

[4] *Department of Ophthalmology Vita-Salute San Raffaele University, 20132 Milan, Italy*

Data Pre-processing

Common preprocessing methods were applied on the raw data obtained from each of the VSDI experiments. First, data from 20 trials were averaged and a pixel-wise detrend was performed using a two-part exponential fit to the data to remove the photo-bleaching effect [1]. Then, $\frac{\Delta F}{F}$ was calculated by subtracting and dividing the exponential fit from each pixel value. Next, we calculated the Z-score by dividing the calculated $\frac{\Delta F}{F}$ value by the standard deviation of the first second, which served as a baseline[2]. For each recording, the spectra of different cortical regions were examined to determine the oscillatory noise frequencies for every individual recording. Generally, the frequencies were 0.39 Hz, 0.67 Hz, 0.78 Hz, 3.3 Hz, and 6.6 Hz. Thus, data were high-pass filtered using an elliptic IIR (order N=8) filter with a cutoff frequency of 0.8 [Hz] to remove slow baseline oscillations, usually arising from breathing.

Supplementary Material Figure 1 *– A raw (df/f) VSD signal in response to 8-grid stimuli given at a rate of 1 [Hz] in the ROI presented in the main text Figure 5.*

The theoretical response curve

The theoretical response curve, which was used by the TSCA, T_{max}, Correlation, and GLM analysis methods as the temporal prior, was a constructer similar to [3]; it was found to be similar to the average experimental response curve recorded from the visual cortex of the rats in the current study, shown in Figure 5. The sensory-evoked theoretical neural responses can be characterized using a set of non-linear parameters $\alpha_i, i \in [1,4]$ that correspond to the response latency, rise time, plateau, and decay time. For each experiment, different ranges for each parameter must be chosen. The authors proposed producing N response curves by randomly selecting N α_is. In order to limit the number of regressors in the model, Singular Value Decomposition (SVD) was performed on different response curves and the first L basis vectors were chosen, such that linear combinations of these basis vectors explained 90% of the shape variance[3]. Muller et al. [4] reported that the stimulus-evoked population response of an awake monkey is a propagating wave, and Gao et al. [5] have observed this phenomenon in the rat visual cortex as well. Thus, a fine center of activity (COA) is expected, with a temporal behavior corresponding to the theoretical response curves used, i.e., a rise time and a relatively fast decay time (delta-function-like behavior), as opposed to a slowly decaying signal (step-function-like behavior). The theoretical response was circularly shifted so that it peaks at 160 msec after stimulus onset, which is the expected theoretical time. The response curve was zero-padded such that its length was the length of the entire recording. Then, it was delayed by 1 second or 0.5 seconds, depending on the frequency the stimuli were given. Each delayed version of the response signal was used as a regressor in GLM, as

the signal to be correlated by the correlation method, and its covariance matrix was estimated by $E[xx^t]$ and was used as C_x in TSCA.

Supplementary Material Figure 2 – A) Temporal behavior of the theoretical response curve and the average temporal response from the experimental data

Data Post-processing

The cortical maps were smoothed using a 2D Gaussian filter with an STD of 0.2 and a 2D median filter of size 3 (3x3 window). Then, all maps were Min-Max normalized, i.e., rescaled to values between 0 and 1.

Retinotopic Map Statistical Measures–a detailed explanation

The retinotopic maps generated by each of the 7 methods (see the main text) were evaluated using both cluster separation metrics and statistical measures. The six statistical measures used for the simulated data-generated maps are presented here. The cluster separation metrics are detailed next.

Mean square error (MSE)

An extensively used measure is the *mean-square-error (MSE)*, defined as [6]

$$(s1)\ \ MSE = \frac{1}{N}\sum_{i=N}^{N}(\hat{I}_i - I_i)^2,$$

where N is the number of pixels in the image, $\hat{I}$ is the generated response, and I is the original (reference) response.

Peak Signal-to-Noise Ratio (PSNR)

$$(s2)\ \ PSNR = 10\log_{10}\frac{\text{peak value}(f)}{MSE}$$

where f are the reference image values.

Contrast-to-Noise Ratio (CNR)

The *contrast-to-noise ratio (CNR)*, defined in [6], gives an objective measure of the useful contrast (the difference of means) between a region of an image feature (target) or ROI and a region of background noise (reference).

$$(s3)\ \ CNR = 10\log_{10}\frac{\mu_t - \mu_r}{\sqrt{\sigma_t^2 + \sigma_r^2}}$$

where μ_t & σ_t^2, μ_r & σ_r^2 are the pixel mean & variance of a target and reference area, respectively, on the image.

Mean Structure Similarity Index (MSSIM)

MSSIM compares the luminance, contrast, and structure of two different images X, Y and is defined as [6][7]:

$$(s4.1)\ \ SSIM(X,Y) = \frac{(2\mu_x\mu_y + C_1)(2\sigma_{xy} + C_2)}{(\mu_x^2 + \mu_y^2 + C_1)(\sigma_x^2 + \sigma_y^2 + C_1)}$$

where μ_x, μ_y are the mean intensity, $\sigma_x, \sigma_y, \sigma_{xy}$ are the standard deviations and covariance, and C_1, C_2 are constants to avoid instability [6]. In order to have an overall quality measurement of the entire image, we used a *mean SSIM index (MSSIM)*, defined as:

$$(s4.2)\ \ MSSIM(X,Y) = \frac{1}{N}\sum_{i=1}^{N} SSIM(X_i, Y_i)$$

where X, Y are the original and reconstructed images, respectively, and N is the number of windows/regions compared in the image.

Correlation Coefficient (CC)

The 2D correlation coefficient (CC), obtained by correlating the reconstructed image and the original images, is an additional statistical measure employed. The CC value gives a general correlation between the two images X, Y and is defined as:

$$(s5)\ \ r = \frac{\sum_{i=1}^{N}(X_i - \mu_x)((Y_i - \mu_y)}{\sqrt{\sigma_x\sigma_y}}$$

where N is the number of pixels in the images.

Correlation Parameter (CP)

To evaluate edge preservation (sharpness), we considered the qualitative measure *Correlation Parameter (CP)* χ proposed in [6], which is the 2-D correlation between ΔX and ΔY, which are the high-pass filtered versions of X, Y, obtained via a 3x3 pixel standard approximation of the Laplacian operator.

$$(s6)\ \ \chi = \frac{\sum_{i=1}^{N}(\Delta X_i - \mu_{\Delta x})((\Delta Y_i - \mu_{\Delta y})}{\sqrt{\sigma_{\Delta x}\sigma_{\Delta y}}}$$

The statistical measures obtained by each method for each SNR (100 repetitions) can be seen here in Supp. Material figure 3. For lower SNR conditions, TSCA and TSCA&GLM showed significantly better MSSIM, CC and CP compared with all other methods, significantly better MSE and PSNR compared with AOF, GLM and Corr and significantly better CNR compared with T_{max} (a multiple comparison t-test with Tukey-HSD, $p<0.05$).

Supplementary Material Figure 3. *Average values of six statistical evaluation methods (MSE, PSNR, CNR, MSSIM, CC and CP) of retinotopic maps generated by the seven analysis methods for simulated cortical responses with decreasing signal-to-noise ratios*

Cluster separation metrics

The cluster separation metrics, DBI and SI, were used for evaluating the retinotopic maps generated by both the simulated and experimental data, with some adjustments (see the main article). A brief mathematical description of each index is given below.

Davies-Bouldin Index (DBI)

The DBI is an internal measure for assessing similarity between clusters ($R_{i,j}$). The higher the DBI is, the more similar the clusters are. Intuitively, the larger the distance between two cluster centroids ($M_{i,j}$) and the smaller the cluster scatters (S_i, S_j) are, the less similar the clusters will be. Thus, the index was defined such that it conserves four basic properties:

1. Cluster similarity is non-negative, i.e., $R_{i,j} \geq 0$.
2. Symmetry, i.e., $R_{i,j} = R_{j,i}$
3. If two clusters i, j are the same distance from a third, k, and one has a larger scatter, it is more similar to cluster k. That is, if $S_j \geq S_k$ and $M_{i,j} = M_{i,k}$, then $R_{i,j} > R_{i,k}$.
4. If two clusters i, j have the same scatter, and one is closer to a third cluster k, then it is more similar to it. That is, if $S_j = S_k$ and $M_{i,j} \leq M_{i,k}$, then $R_{i,j} > R_{i,k}$.

given an n-dimensional feature vector X_l assigned to cluster C_l, with size T_i, and A_l be its centroid; the similarity between two clusters is defined as:

$$(s7)\quad R_{i,j} = \frac{S_i + S_j}{M_{i,j}}$$

where $S_i = \left(\frac{1}{T_i}\sum_{k=1}^{T_i}|X_k - A_i|^p\right)^{1/p}$ and $M_{i,j} = \left(\sum_{k=1}^{n}\left|X_{k,i} - X_{k,j}\right|^q\right)^{1/q}$. Here, $n = 2$; the feature vector of each cluster is two Euclidean coordinates. We expected a higher similarity between responses from neighboring stimuli and a low similarity between responses from stimuli that are far apart. From $R_{i,j}$, $D_i = \max_{j \neq i} R_{i,j}$. D_i is the worst-case scenario, that is, the two clusters that are the most similar. Then, the DBI (Davis-Bouldin Index) is defined as $DBI = \frac{1}{N}\sum_{i=1}^{N} D_i$, that is, the average D_i of all N clusters. From its definition, a high DBI implies a high similarity, i.e., a poor separation and a low DBI implies good separation.

Silhouette Index (SI)

The silhouette index, $S(i)$, measures how similar an object is to its own cluster, compared to other clusters. It ranges from [-1,1], where if $S(i)$ approaches 1, the data point i is appropriately clustered. However, if $S(i)$ approaches -1, point i is inappropriately clustered. If $S(i)$ approaches 0, point i is on the border of 2 clusters. It is an internal measure that gives a score to each point in the data set by taking into consideration the point's average distance to its own cluster and the minimal average distance to a different cluster.

For each point i in cluster C_i, we can calculate:

1. The within cluster dissimilarity $a(i)$, defined as $a(i) = \frac{1}{|c_i|-1}\sum_{j \in C_i, j \neq i} d(i,j)$, where $|c_i|$ is the number of elements in cluster i , and $d(i,j)$ is a distance metric (the Euclidean distance here).

2. The between cluster dissimilarity $b(i)$, is defined as $b(i) = \min_{k \neq i} \frac{1}{|c_k|}\sum_{j \in C_k} d(i,j)$.

$a(i)$ is the mean distance of data point i to all points in its cluster C_i. $b(i)$ is the smallest mean distance of data point i to all points in all other clusters (excluding C_i). Ideally, $a(i) \ll b(i)$.

We the Silhouette index is then defined as $(s8)\quad S(i) = \begin{cases} \frac{b(i)-a(i)}{\max\{b(i),a(i)\}} & , \ |c_i| > 1 \\ 0 & , \ |c_i| = 1 \end{cases}$.

As described in the main text, we calculated the adjusted silhouette and the DBI indices by penalizing each cluster. The cluster penalty is calculated by:

$$(s9.1)\quad Cluster\ Penalty = \frac{|Cluster\ size - Expected\ size|}{Expected\ size}$$

Since all stimuli are the same size and close enough to assume an equal CMF, we expect the centers of activity generated by each stimulus to be the same size. The map penalty is calculated by:

$$(s9.2)\ \ Map\ Penalty = \frac{|Number\ of\ Clusters - Expected\ number\ of\ Clusters|}{Expected\ number\ of\ clusters}$$

Since the experimental protocol is known, we know the expected number of centers of activity, i.e., clusters. The adjusted DBI and Silhouette are calculated as follows:

$$(s10)\quad adjusted\ DBI = DBI + avg(Cluster\ Penalty) + Map\ Penalty$$

$$(s11)\quad adjusted\ Silhouette_i = Silhouette_i - (Cluster\ Penalty + Map\ Penalty)$$

Combining Methods

All the methods used here have an output that is a 3D map of scores per pixel per stimulation, i.e., the first two dimensions are the size of the frame (representing all the pixels in the frame) and the third dimension's size is the number of stimulations shown in an experiment. A way to combine methods (e.g., TSCA with AOF) is to perform a weighted average of the scores given to each pixel (to each stimulus) by different methods. Supp. Material Figure 4A shows the six statistical performance measures and the clustering separation measures obtained by performing a weighted average of the TSCA and AOF scores. Likewise, Figure 4B shows the combination of TSCA and Corr. As the weight of TSCA increases, the statistical and cluster separation measures improve, indicating that TSCA by itself performs better than combining it with other methods.

Of all the six methods, GLM can be used to give a score for every pixel (as explained in the main text), but it can also be used as a denoising step (as explained in the main text and in [3]). We denoised the signal using GLM and then performed the remaining five methods, and qualitatively observed that it improves the maps generated, however does not improve the quantitative measures significantly. Since TSCA has proven to be the best method, combining it with GLM improved the generated maps slightly.

Supplementary Material Figure 4. *The six statistical measures and two cluster separation measures obtained for a simulation in -10dB for a combination by weighted averaging of A) TSCA and AOF and B) TSCA and Corr.*

Optimal parameters for each method

Each of the analysis methods relies on free parameters to be chosen by the user (see the main text). These parameters usually provide thresholds for each algorithm to decide whether a pixel responded to a specific condition and to quantify the magnitude of the response (score). Methods that attempt to denoise the signal also have the noise source parameters (e.g., the frequencies of the oscillatory noises) to be chosen. In order to properly evaluate the six different methods across several experiments, we used the same value for these parameters across all experiments. The constant parameters were chosen following a trial-and-error optimization process.

Supplementary Material Table 1

Optimal parameters chosen for analyzing the simulated data.

Method	Parameters
**TSCA**	C_x: covariance matrix of a Gaussian response curve with s.t.d=1<br>C_y: identity matrix and Toeplitz matrices of oscillatory noises<br>Toeplitz matrices of oscillatory noises:<br>• Frequencies – 0.67[Hz], 3[Hz]<br>• Bandwidth – 0.1 [Hz]<br>• # of harmonics = 3<br>• Harmonics weights: 1, 0.1, 0.1<br>Gammas:<br>• Signal: 1<br>• White Noise: -0.25<br>• Oscillatory Noise: -0.05
**T_{max}**	Reference signal to cross-correlate pixel-wise: A Gaussian response curve with s.t.d=1<br>Threshold of cross-correlation values r: $\bar{r} + 0.2 * std(r)$
**AOF**	Frames to average: $t_{res} - 25$ until $t_{res} + 25$ (25 [msec] before and after the response peaked)
**Corr**	(Delayed) Reference signal to correlate pixel-wise: A Gaussian response curve with s.t.d=1
**GLM**	(Delayed) Reference signals as regressors: A Gaussian response curve with s.t.d=1<br>Oscillatory noise frequencies– 0.67[Hz], 3[Hz]
**MPT**	Absolute threshold value: 0.2<br>Percentile threshold of all peak values: 0 (i.e., all peak values)<br>Latency of response (peak value): 100[msec] before and after the peak response<br>Settle (of each pixel $p(t)$):<br>• Threshold value: $\overline{p(t)} + 1 * std(p(t))$<br>• Time until settle: 30[msec] from the end of the condition

Supplementary Material Table 2

Optimal parameters chosen for each method (experimental data)

Method	Parameters
**TSCA**	C_x: covariance matrix of the theoretical response curve (Figure 1)<br>C_y: identity matrix and Toeplitz matrices of the oscillatory noises<br>Toeplitz matrices of the oscillatory noises:<br>• Frequencies – chosen independently for each experiment<br>• Bandwidth – 0.1 [Hz]<br>• # of harmonics = 3<br>• Harmonics weights: 1, 0.1, 0.1<br>Gammas:<br>• Signal: 1<br>• White Noise: -0.25<br>• Oscillatory Noise: -0.05
**T_{max}**	Reference signal to cross-correlate pixel-wise: the theoretical response curve (Supp. Material Figure 1)<br>Threshold of the cross-correlation values r: average of values ($\bar{r}$)
**AOF**	Frames to average: 10-30 (100-300[msec] from the stimulus onset)
**Corr**	(Delayed) Reference signal to correlate pixel-wise: the theoretical response curve (Supp. Material Figure 1)
**GLM**	(Delayed) Reference signals as regressors: the theoretical response curve (Supp. Material Figure 1)<br>Oscillatory noise frequencies – chosen independently for each experiment
**MPT**	Absolute threshold value: 0.2<br>Percentile threshold of all peak values: 0 (i.e., all peak values)<br>Latency of response (peak value): 100 – 300 [msec] from the stimulus onset<br>Set (for each pixel $p(t)$):<br>• Threshold value: $\overline{p(t)} + 3 * std(p(t))$<br>• Time until it is set: 50 [msec] from the end of each condition

Additional Experimental VSDI Data

Supplementary Material Figure 5. *Cortical responses to 8 location stimuli from the same experiment (animal), with 4 different trials. Only pixels with values above the 90th percentile of all the pixels' maximum scores are shown in color. The scale bar shown in the top left panel is 1mm. The cortical orientations are seen in the bottom right figure, along with the expected cortical response (A – anterior, P – posterior, M – medial, and L – lateral). Each color (hue) represents the cortical response location of the matched retinal stimuli.*

Supplementary Material Figure 6. *Retinotopic maps generated in response to 3 vertical bars (A) and 4 location stimuli (B). Only pixels with values above the 90th percentile of all the pixels' maximum scores are denoted in color. The scale bar shown in the top left panel is 1mm. The cortical orientations are seen in the bottom right figure, along with the expected cortical response (A – anterior, P – posterior, M – medial, and L – lateral). Each color (hue) represents the cortical response location of the matched retinal stimuli.*

Supplementary Material Figure 5 presents retinotopic maps generated following 4 repetitions of the same experimental sessions in the same animal. Qualitative evaluation suggests that TSCA and the combined TSCA and GLM generated a stable and consistent retinotopic map. In contrast, other analysis methods showed a gradual deterioration in map quality, arising from both physiological and physical (e.g., dye bleaching) factors, causing a decreased signal-to-noise ratio as the experimental sessions progressed.

Supplementary Material Figure 6 presents the results obtained by each of the 7 methods investigated in this research for 3 bars and 4 location grid stimuli, both are significantly larger and farther apart than the 8 and 9 bars presented in the main text. In addition, the noise level was low in these specific experiments. All methods apart from T_{max} and MPT were able to localize the responses to each stimulus and thus well separated retinotopic maps were generated.

All the software code was written in MATLAB R2019b and is publicly available in "github": https://github.com/oricarmi/VSDI_MATLAB_COMPARISON.git

[1] T. Fekete, D. B. Omer, K. O'Hashi, A. Grinvald, C. van Leeuwen, and O. Shriki, "Critical dynamics, anesthesia and information integration: Lessons from multi-scale criticality analysis of voltage imaging data," *Neuroimage*, 2018.

[2] A. Gross, N. H. Ivzan, N. Farah, and Y. Mandel, "High-resolution VSDI retinotopic mapping via a DLP-based projection system," *Biomed. Opt. Express*, 2019.

[3] A. Reynaud, S. Takerkart, G. S. Masson, and F. Chavane, "Linear model decomposition for voltage-sensitive dye imaging signals: Application in awake behaving monkey," *Neuroimage*, 2011.

[4] L. Muller, A. Reynaud, F. Chavane, and A. Destexhe, "The stimulus-evoked population response in visual cortex of awake monkey is a propagating wave," *Nat. Commun.*, 2014.

[5] X. Gao, W. Xu, Z. Wang, K. Takagaki, B. Li, and J. Y. Wu, "Interactions between two propagating waves in rat visual cortex," *Neuroscience*, 2012.

[6] H. M. Salinas and D. C. Fernández, "Comparison of PDE-based nonlinear diffusion approaches for image enhancement and denoising in optical coherence tomography," *IEEE Trans. Med. Imaging*, 2007.

[7] Z. Wang, A. C. Bovik, H. R. Sheikh, and E. P. Simoncelli, "Image quality assessment: From error visibility to structural similarity," *IEEE Trans. Image Process.*, 2004.
