# Supplementary figures and images for: Evaluation and Optimization of Methods for Generating High-Resolution Retinotopic Maps Using Visual Cortex Voltage-Sensitive Dye Imaging
Source: Front Cell Neurosci. 2021 Sep 21;15:713538. doi: 10.3389/fncel.2021.713538 (PMC8490879; doi:10.3389/fncel.2021.713538)

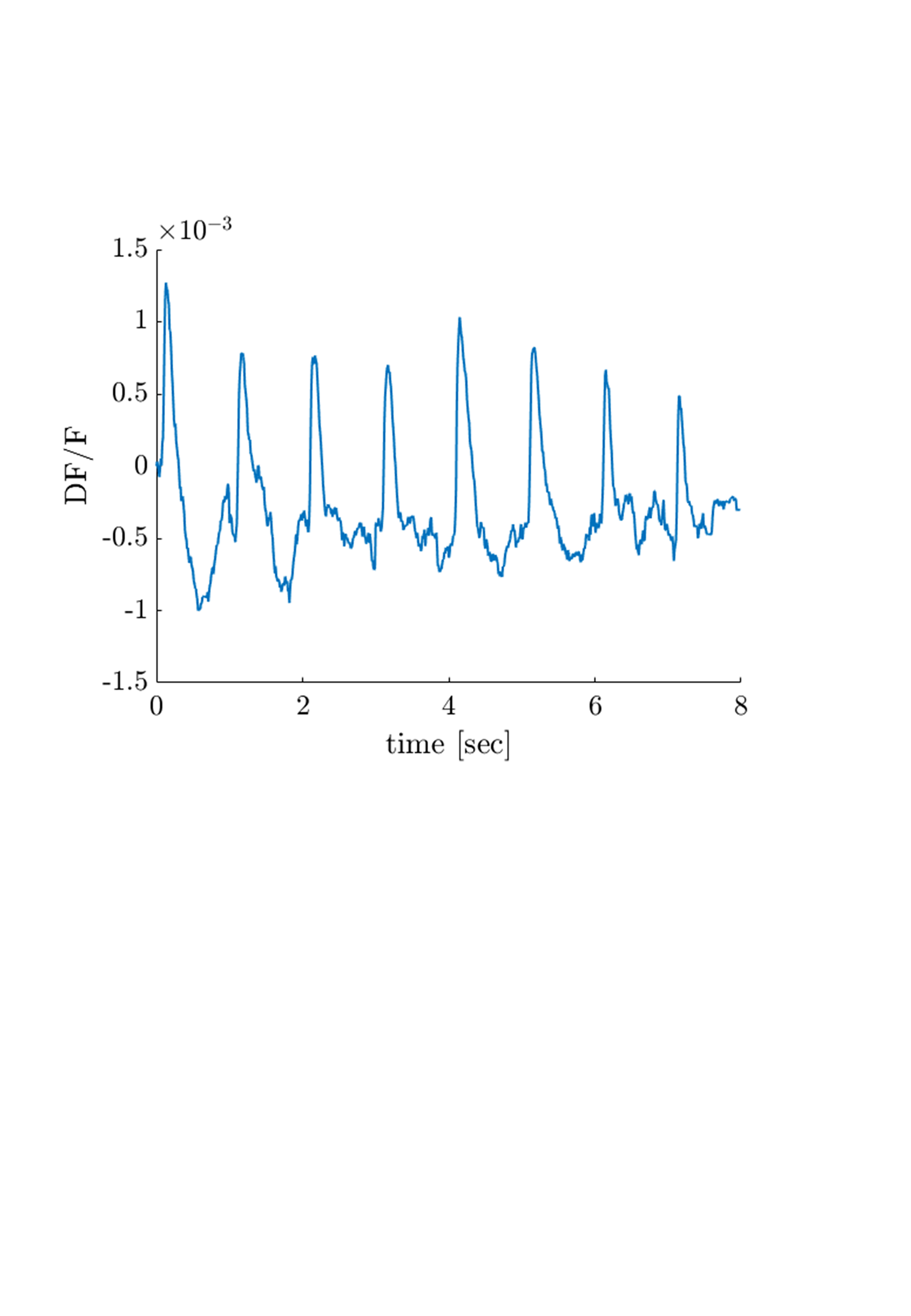

Supplement: Supplementary file 2 [file Image_1.TIF]

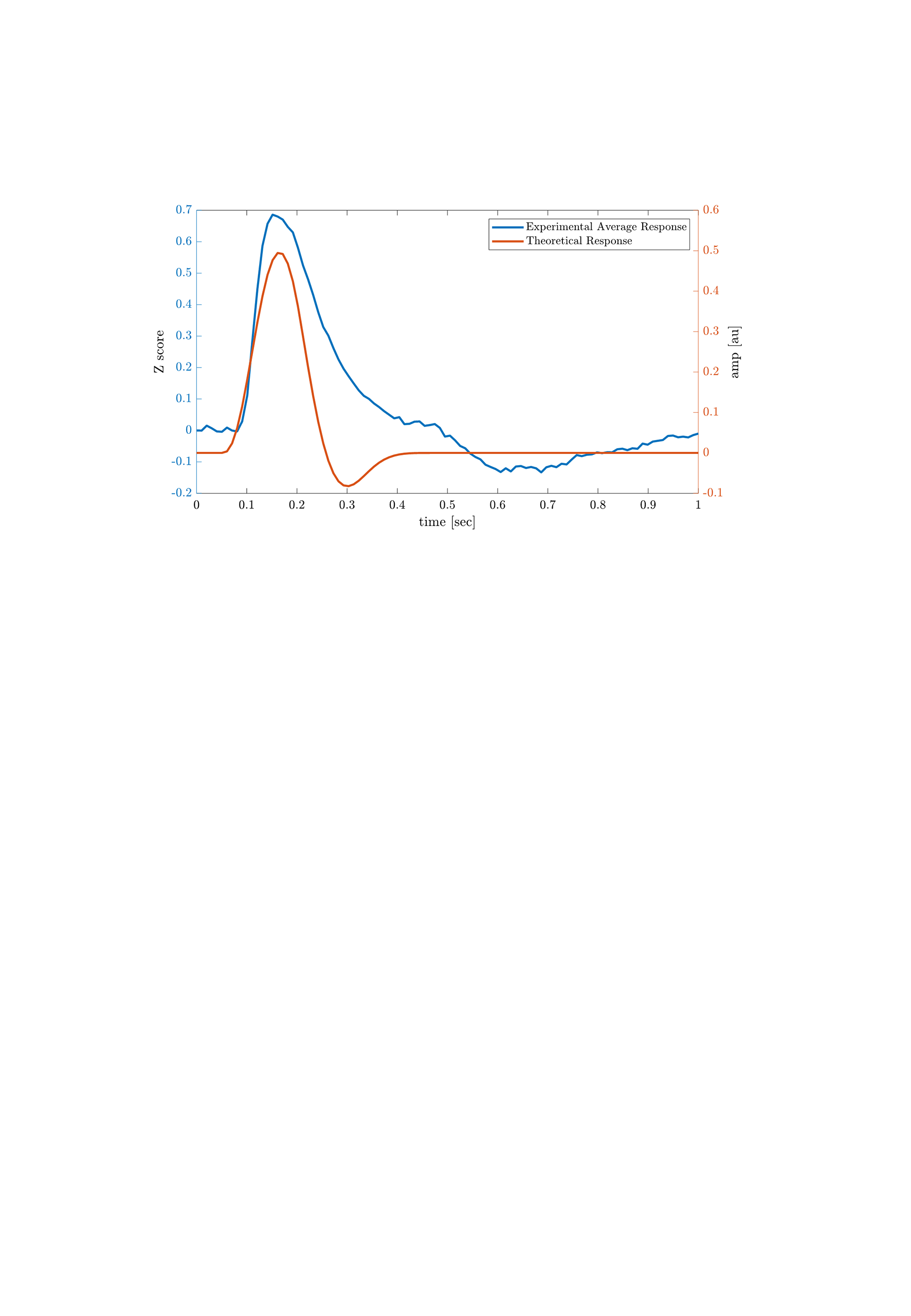

Supplement: Supplementary file 3 [file Image_2.TIF]

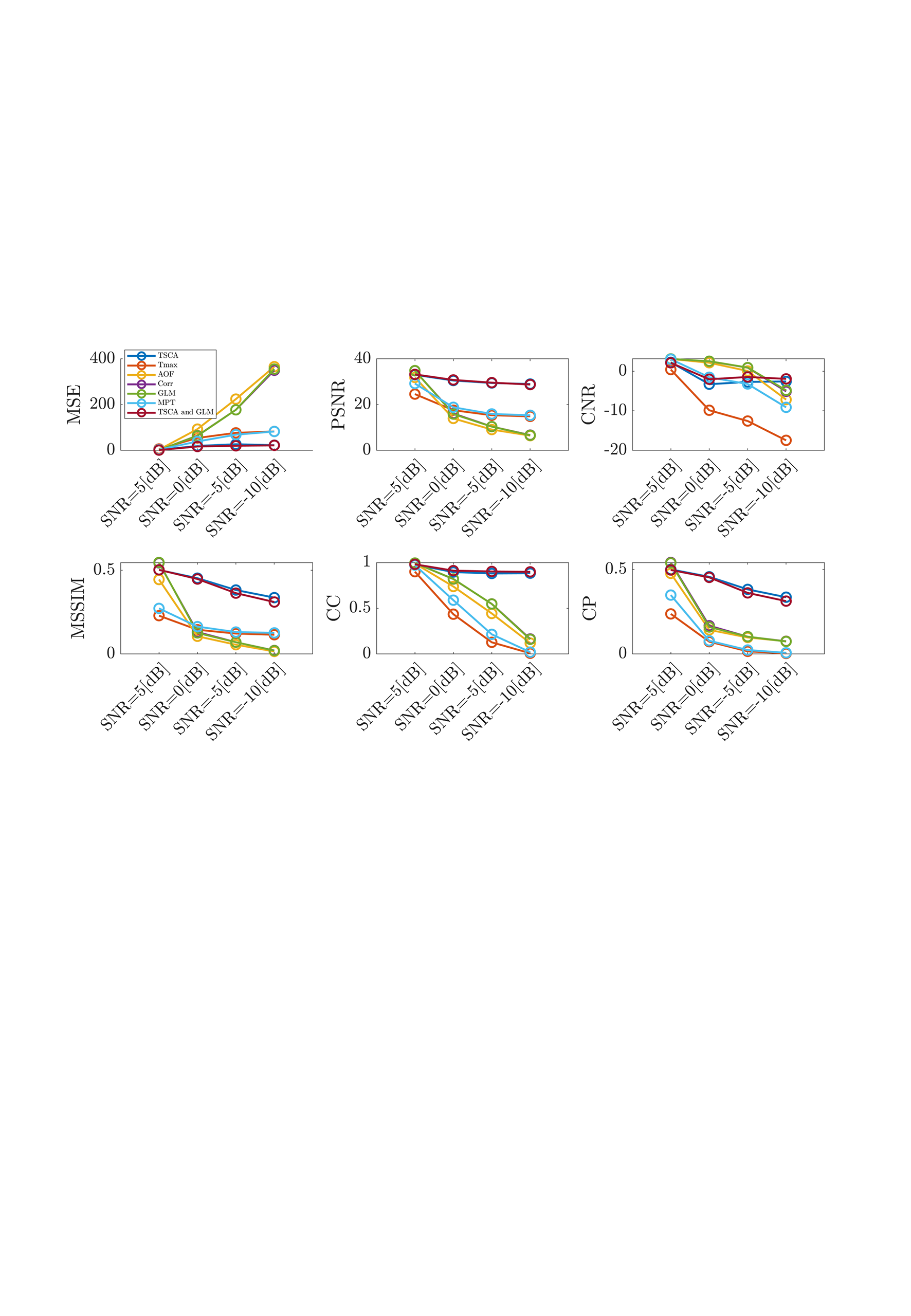

Supplement: Supplementary file 4 [file Image_3.TIF]

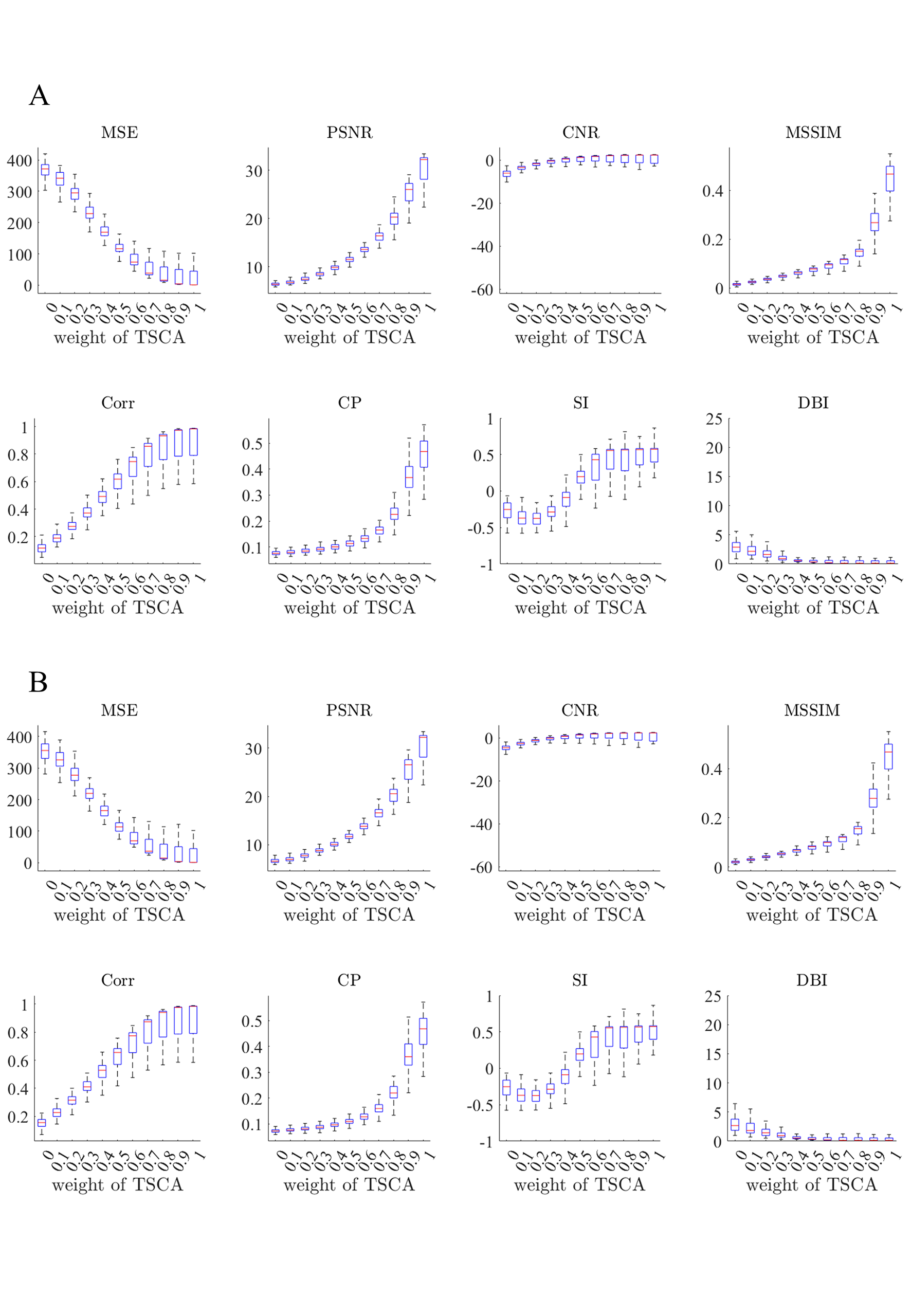

Supplement: Supplementary file 5 [file Image_4.TIF]

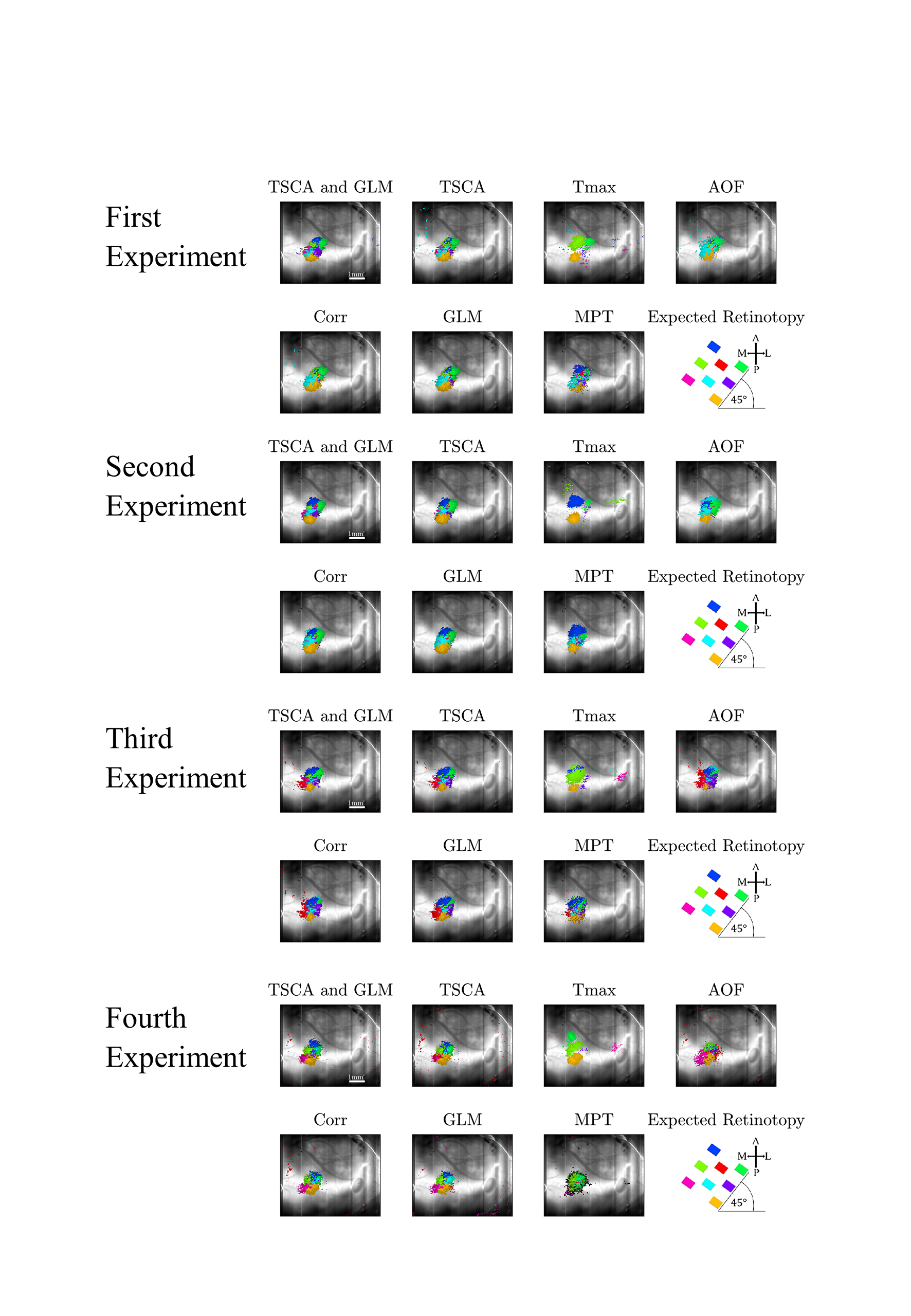

Supplement: Supplementary file 6 [file Image_5.TIF]

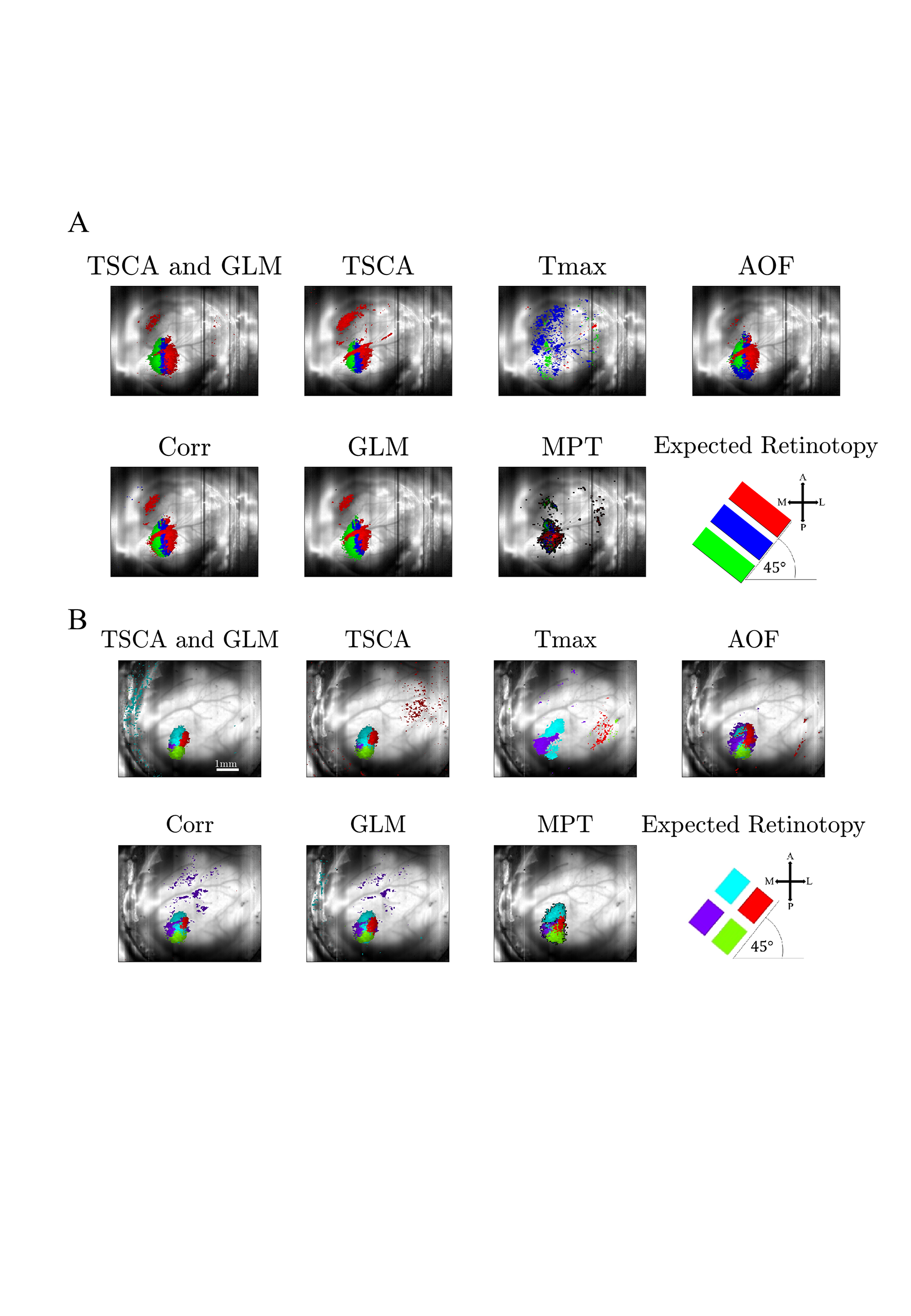

Supplement: Supplementary file 7 [file Image_6.TIF]
